# Bioinformatic Analysis Identifies Biomarkers and Treatment Targets in Primary Sjögren's Syndrome Patients with Fatigue

**DOI:** 10.1155/2022/7697558

**Published:** 2022-01-15

**Authors:** Guangshu Chen, Li Che, Xingdong Cai, Ping Zhu, Jianmin Ran

**Affiliations:** ^1^Department of Endocrinology, Guangzhou Red Cross Hospital, Jinan University, Guangzhou 510220, China; ^2^Department of Pulmonary and Critical Care Medicine, The First Affiliated Hospital of Jinan University, Guangzhou 510630, China

## Abstract

We aim to identify the common genes, biological pathways, and treatment targets for primary Sjögren's syndrome patients with varying degrees of fatigue features. We select datasets about transcriptomic analyses of primary Sjögren's syndrome (pSS) patients with different degrees of fatigue features and normal controls in peripheral blood. We identify common differentially expressed genes (DEGs) to find shared pathways and treatment targets for pSS patients with fatigue and design a protein-protein interaction (PPI) network by some practical bioinformatic tools. And hub genes are detected based on the PPI network. We perform biological pathway analysis of common genes by Gene Ontology (GO) terms and Kyoto Encyclopedia of Genes and Genomes (KEGG) pathway. Lastly, potential treatment targets for pSS patients with fatigue are found by the Enrichr platform. We discovered that 27 DEGs are identified in pSS patients with fatigue features and the severe fatigued pSS-specific gene is RTP4. DEGs are mainly localized in the mitochondria, endosomes, endoplasmic reticulum, and cytoplasm and are involved in the biological process by which interferon acts on cells and cells defend themselves against viruses. Molecular functions mainly involve the process of RNA synthesis. The DEGs of pSS are involved in the signaling pathways of viruses such as hepatitis C, influenza A, measles, and EBV. Acetohexamide PC3 UP, suloctidil HL60 UP, prenylamine HL60 UP, and chlorophyllin CTD 00000324 are the four most polygenic drug molecules. PSS patients with fatigue features have specific gene regulation, and chlorophyllin may alleviate fatigue symptoms in pSS patients.

## 1. Introduction

Primary Sjögren's syndrome (pSS) is an all-body autoimmune disease that mainly affects middle-aged women [1]. The main clinical feature of the disease is dryness of the mouth and eyes, and the pathophysiology is characterized by focal lymphocyte infiltration in exocrine glands [2, 3]. Fatigue is commonly seen in pSS patients as an extraglandular manifestation and closely links with poor life quality [4–6]. Fatigue affects approximately 70% of pSS patients [7, 8]. Normally, fatigue and depression are considered manifestations of psychological disorders and interact with physical pain and discomfort, which creates a vicious cycle. Fatigue in pSS is induced and regulated by genetic and molecular mechanisms, with the innate immune system playing an important role in the production of fatigue [9–11]. Although pSS always comes with fatigue, not all patients exhibit fatigue, which provides a good model for exploring the underlying biological mechanisms.

High-throughput methods play an increasingly essential role in biology spheres, and microarray data analysis highlights its advantage in large-scale analysis of gene expression among high-throughput applications [12, 13]. Former studies [14, 15] have shown the high-throughput sequencing analysis result for pSS patients with fatigue features but do not offer further analysis based on varying degrees of fatigue. This study tries to present characteristic genes and biological pathways in pSS patients with manifestations of fatigue, as well as drugs of potential benefit.

The GSE66795 dataset from the GPL10558 platform on the GEO database is selected for gene expression of pSS with fatigue. The GSE66795 dataset was first identified for differentially expressed genes (DEGs) in pSS patients with different levels of fatigue, and based on the coexpressed genes, further analyses including Gene Ontology (GO) terms and Kyoto Encyclopedia of Genes and Genomes (KEGG) pathway are performed to understand the biological process. The top ten target genes from the protein-protein interaction (PPI) network will be obtained to identify potential drugs that may alleviate fatigue in pSS patients.

## 2. Materials and Methods

### 2.1. Dataset Collection

We search “Primary Sjögren's syndrome” and “fatigue” in the GEO database [16] and select the dataset (GSE66795) demonstrating gene expression in pSS patients with varying degrees of fatigue characteristics and normal controls. The GSE66795 dataset is extracted from the GPL10558 platform (Illumina HumanHT-12 V4.0 expression microbead chip) for RNA sequence analysis. The data of GSE66795 is obtained from the UK registry of primary Sjögren's syndrome. It includes whole genome microarray profiles of pSS patients with varying degrees of fatigue characteristics and normal controls in peripheral blood. One hundred and thirty-one patients with pSS are involved, including 21 patients with mild fatigue, 74 patients with moderate fatigue, 36 patients with severe fatigue, and 29 normal controls.

### 2.2. Differential Expression Analysis

Differential expression analysis is performed using the online analysis tool GEO2R; gene expression profiles of pSS patients with mild, moderate, and severe fatigue were compared with normal controls separately to identify DEGs. *P* values and adjusted *P* values are calculated using *t*-tests. Genes with the following criteria were retained for each sample: (1) log2-fold change (log2FC) absolute value greater than 1 and (2) adjusted *P* value less than 0.05. After identifying DEGs in pSS patients with varying degrees of fatigue, the online website (https://www.xiantao.love/gds) is used to plot a Venn diagram.

### 2.3. Gene Ontology and Pathway Discovery in Gene Set Enrichment Analysis

Gene set enrichment analysis is used to understand the general biological function and the chromosomal location of a gene [17]. For gene product annotation, the terms of Gene Ontology (GO) are used, including biological process (BP), molecular function (MF), and cellular component (CC) [18]. The Kyoto Encyclopedia of Genes and Genomes (KEGG) pathways are commonly used to describe metabolic pathways [19]. GO terms and KEGG pathways were gotten through the platform Enrichr (https://amp.pharm.mssm.edu/Enrichr/) based on the DEGs [20].

### 2.4. Protein-Protein Interaction (PPI) Network

The information generated from the PPI network improves the understanding of protein function [21]. PPI networks are made by STRING (https://string-db.org/) after inputting the common DEGs. We analyze PPIs through Cytoscape (https://cytoscape.org/) to further present the network and identify target genes.

### 2.5. Transcription Factor- (TF-) Gene Interactions

We use NetworkAnalyst (https://www.networkanalyst.ca/) to identify interactions of TF-genes with DEGs [22]. NetworkAnalyst plays a comprehensive network platform for gene expression across a wide range of species and enables them to be subjected to a meta-analysis [23].

### 2.6. Identification of Potential Treatment Targets

Identification of drug molecules is a vital component of genomics research. We input the DEGs in the Drug Signature Database (DSigDB). Then, we get the designed drug molecules, which may have promising clinical application. DSigDB is obtained through the Enrichr (https://amp.pharm.mssm.edu/Enrichr/) platform. Enrichr is primarily used as an enrichment analysis platform, providing extensive visual details of the common functions of inputted genes [24].

## 3. Results

### 3.1. DEG Identifications

**Figure 1:**
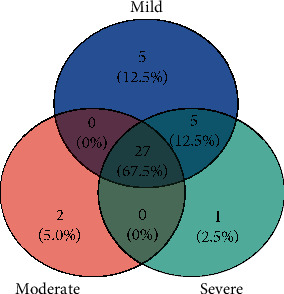
A Venn diagram represents common DEGs. Twenty-seven genes are found common among the 40 DEGs of pSS patients with varying degrees of fatigue. The common DEGs accounted for 67.5% out of a total of 40 DEGs.

We use the GSE66795 dataset to identify the DEGs of pSS with fatigue. 37, 29, and 33 DEGs are obtained for pSS with mild, moderate, and severe fatigue, respectively. The collected DEGs are further compared by using the online website (https://www.xiantao.love/gds) for gathering common genes in pSS with varying degrees of fatigue. And 27 (OAS1, OAS2, GBP1, IRF7, EIF2AK2, IFIT2, USP18, SAMD9L, HES4, IFI44L, SERPING1, IFIT3, IFITM3, IFI6, XAF1, MX1, OASL, OTOF, HERC5, LY6E, EPSTI1, OAS3, ISG15, IFIT1, RSAD2, IFI44, and IFI27) common DEGs are identified. The specific genes to pSS with mild fatigue are DDX60, IFIH1, GBP5, LAP3, and TIMM10. The specific genes to pSS with moderate fatigue are HLA-DRB4 and HLA-DRB6, and that to pSS with severe fatigue is RTP4. The Venn diagram (Figure 1) shows that common DEGs accounted for 67.5% out of a total of 40 DEGs.

### 3.2. GO Terms and KEGG Pathways

**Figure 2:**
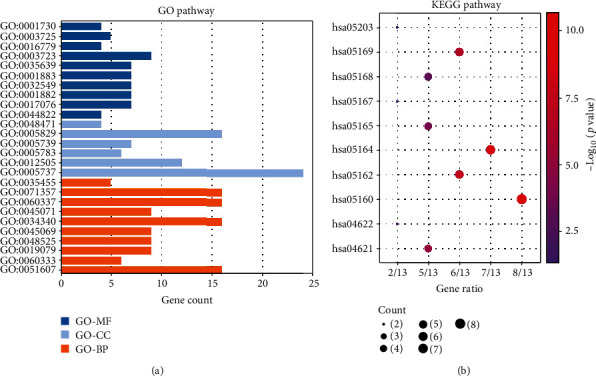
(a) The result of biological process-, molecular function-, and cellular component-associated GO terms. (b) The result of KEGG pathway analysis.

We analyzed 27 common DEGs for both GO and KEGG pathways. Both of the results are taken from the top 10 GO entries. GO terms in Table 1 suggest that DEGs are mainly localized in the mitochondria, endosomes, endoplasmic reticulum, and cytoplasm. They are involved in the biological processes of interferon action on cells and cellular defense against viruses. And the molecular functions are mainly engaged in the process of RNA synthesis. KEGG pathways in Table 2 suggest that the DEGs of pSS with fatigue are involved in the signaling pathways of viruses such as hepatitis C, influenza A, measles, and EBV. Both are seen in Figures 2(a) and 2(b).

### 3.3. Identification of Hub Genes by PPI Networks

**Figure 3:**
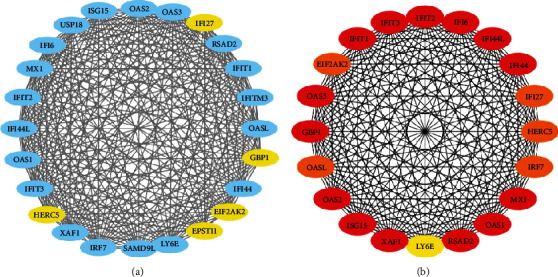
(a) Protein-protein interaction (PPI) network for DEGs of patients with varying degrees of fatigue. Nodes in blue color indicate common DEGs with the highest connection degree value, and edges specify the interconnection in the middle of two genes. The network involves 24 nodes and 552 edges. (b) The top 20 genes are detected from the PPI network of common DEGs. The nodes in red color represent 14 genes with the highest degree value which are MX1, IFIT1, ISG15, RSAD2, IFI44L, IFI44, IFIT3, OAS2, OAS1, IFI6, XAF1, IFIT2, GBP1, and OAS3. Based on the topological analysis, the degree value of these 14 genes is both 23.

We put common DEGs into the STRING website, and the files generated after analysis are further entered into Cytoscape software for visual analysis. PPI networks are designed to detect hub genes for identifying drug molecules for pSS with fatigue. PPI networks involve 24 nodes and 552 edges, which are shown in Figure 3(a). We present the top 20 genes in Figure 3(b) and Table 3.

### 3.4. TF-Gene Interactions

**Figure 4:**
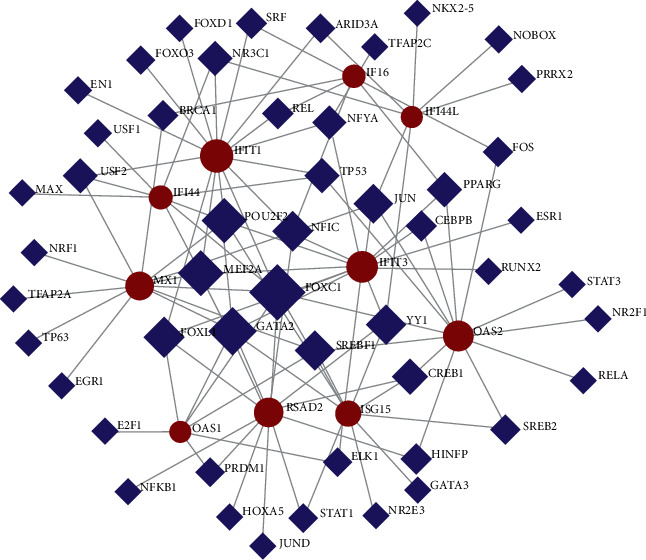
Network for TF-gene interaction with common DEGs. The highlighted 10 red color nodes represent the common genes, and other nodes represent TF-genes. The network has 60 nodes and 108 edges.

The interactions of TF and genes are shown in Figure 4. The network has 60 nodes and 108 edges. Sixteen TF-genes regulate IFIT1, and IFIT3 is handled by 14 TF-genes. The network involves 60 TF-genes. Figure 4 shows the network of TF-gene interactions.

### 3.5. Identification of Drug Candidates

**Figure 5:**
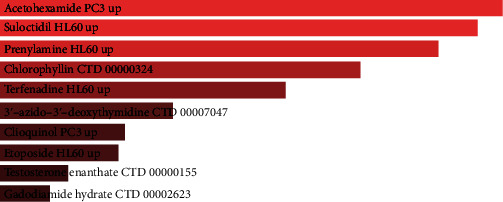
Acetohexamide PC3 UP, suloctidil HL60 UP, prenylamine HL 60 UP, and chlorophyllin CTD 00000324 are the four most polygenic drug molecules that connect with the top 10 hub genes.

We identify drug molecules for the top 10 hub genes on the Enrichr platform. We collect drug candidates judged on adjusted *P* values. The analysis reveals that acetohexamide PC3 UP, suloctidil HL60 UP, prenylamine HL 60 UP, and chlorophyllin CTD 00000324 are the four most polygenic drug molecules that interact with genes.  Figure 5 and Table 4 present the drug candidates in DSigDB.

## 4. Discussion

Fatigue is an annoying experience that means physical and mental tiredness [25]. Mengshoel et al. [26] reveal that most pSS patients literally suffer from fluctuating fatigue out of control regardless of their health condition. Fatigue has a significant influence on patients' daily life, and patients must adapt to their behavior and lives. Although the underlying mechanisms are still unclear, former studies take depression and pain as the prominent factors associated with fatigue [5, 27]. Currently, growing evidence suggests that fatigue has a molecular and genetic basis on its production and regulation. Therefore, most scholars view fatigue as a biological and brain phenomenon [9–11].

IL-1*β* tends to increase rapidly secreted from macrophages to activate the immune system when meeting tissue injury or infection. IL-1*β* plays its role by binding with the IL-1 receptor coming with the downstream of IL-1 response [28]. Then, immune and inflammation systems are activated, which induce the behavior of disease, with fatigue being involved as an important component [29]. All these inflammatory signaling pathways go on working and turn fatigue into a chronic state. In the brain, IL-1 *β* signaling pathways may explain the ultimate pathway of fatigue [30, 31], and IL-1 blocker treatment may effectively release fatigue [32, 33]. Thus, fatigue and other unpleasant mood in those patients with autoimmune disease not only should be understood by the unfortunate development of chronic illness but also may be related to some signaling pathways and activation of genes that regulate the mood in the cerebral system.

Genome-wide association analysis of pSS patients has been conducted, and a gene (RTP4) is identified as highly relevant. Similarly, we confirm that RTP4 is highly expressed in pSS patients with severe fatigue through bioinformatic analysis, suggesting that this gene is critical in the mechanisms of fatigue. RTP4 encodes a protein associated with the expression of opioid receptors on the cell surface. These receptors are also expressed in the lymph system and pain-regulated pathways in the brain [34]. However, the former study did not stratify pSS based on the degree of fatigue, and it is unclear which degree of fatigue expresses the RTP4 gene. Our study finds that pSS patients with severe fatigue specifically express the RTP4 gene, providing clues for further studies on the genomics of fatigue features in pSS patients.

OAS1, a coexpressed gene for pSS in our study, has been established in previous studies as a risk locus of pSS and impacts the flaw of virus clearance because of the altering response of IFN [35]. Our gene pathway analysis points out that DEGs for pSS with fatigue are mainly localized intracellularly and involved in signaling pathways of common viruses in the respiratory and digestive tracts, suggesting that pSS is a systemic disease with an uncertain etiology and that viral infection may be a predisposing factor.

Fatigue always accompanies pSS patients, but it is hard work to manage these bad feelings [36]. The clinical practice guidelines (CPG) committee emphasizes the many causes of fatigue in pSS; therefore, the comprehensive evaluation for diagnosis is essential. So far, the treatment for fatigue in pSS with solid recommendation is mere taking exercise, which is also practical in other autoimmune diseases [37]. In America, hydroxychloroquine (HCQ) is the most widely used drug therapy for pSS with fatigue, but the recommendation strength is not strong enough [34]. It is not recommended to release fatigue in pSS using dehydroepiandrosterone (DHEA) [34]. Both the tumor necrosis factor inhibitor is discouraged for the treatment of fatigue in pSS [38, 39]. Our bioinformatic study reveals that besides chloroquine and testosterone drugs that help improve fatigue, chlorophyllin, the sulphonylurea hypoglycaemic drug acetylhexane, and the antiallergic drug terfenadine may have improved fatigue in pSS. However, chloroquine and testosterone are not strongly recommended as we mentioned before. Acetohexamide has been discontinued in the American market due to its significant hypoglycaemic risk. Terfenadine is not suitable for long-term use since its central depression as an antiallergy drug. And chlorophyllin appears to hold some promise for reducing fatigue in pSS.

Chlorophyll is an ingredient of the derifil drug which is available as an over-the-counter medicine [40]. And chlorophyllin, obtained by hydrolyzing chlorophyll to remove phytyl alcohol, is a water-soluble derivative. Chlorophyll has been shown to exert its anticancer properties by playing a role as an antioxidant [41], a CYP inhibitor [42], an apoptosis inducer [43], a phase II enzyme stimulator [44], and a carcinogen transport modulator [45]. Currently, COVID-19 has swept the world and may last for a long time because of its rapid mutation. Almost 5,000,000 people have died in this epidemic [46], and the reduction of lymphocytes in COVID-19 patients is considered an important risk factor for poor prognosis [47–49]. Recent studies suggest that the chlorophyll derivative sodium copper chlorophyllin (SCC) may improve survival in critically ill COVID-19 patients by increasing the total number of lymphocytes [50]. Increasing consumers choose dietary chlorophyll which is derived from SCC for diet supplements for the sake of keeping healthy [51, 52]. Dietary chlorophyll is safe and has been shown to have a higher absorption rate in the human body, which may trigger ionic compound chelation [53, 54]. Zeng et al. [55] cognize one functional food called barley grass powder which is rich in chlorophyll, and other nutrients can effectively alleviate fatigue in chronic patients. The mechanism of chlorophyll's role in relieving fatigue in pSS patients is unclear. It may be related to the nature of the hepatic enzyme inhibitors that increase the concentrations of immunosuppressant like hydroxychloroquine, which has better control of fatigue. And the capacity of scavenging the oxygen radical as an antioxidant may somewhat improve the fatigue of body.

We have identified gene expression profiles in peripheral blood specific to pSS with fatigue characteristics. The analysis of identified DEGs and pathways in this study will deepen our understanding of the essence of fatigue in pSS. The discovery that chlorophyllin may improve fatigue symptoms provides a theoretical basis for better improving the quality of life in pSS patients. And a preprint has previously been published [56].

## Figures and Tables

**Table 1:** Top 10 GO pathways and their corresponding *P* values and genes.

GO	GO_ID	Description	P-value	Genes
MF	GO:0001730	2'-5'-oligoadenylate synthetase activity	1.305e-12	OAS2 OAS3 OASL OAS1
	GO:0003725	double-stranded RNA binding	1.225e-09	OAS1 OAS2 EIF2AK2 OAS3 OASL
	GO:0016779	nucleotidyltransferase activity	2.993e-06	OAS1 OAS3 OAS2 OASL
	GO:0003723	RNA binding	2.089e-04	EIF2AK2 OAS1 OAS3 HERC5 IFIT2 IFIT3 IFIT1 OASL OAS2
	GO:0035639	purine ribonucleoside triphosphate binding	8.781e-03	EIF2AK2 MX1 OAS1 OAS3 GBP1 OASL OAS2
	GO:0001883	purine nucleoside binding	9.156e-03	EIF2AK2 MX1 OAS1 OAS3 GBP1 OASL OAS2
	GO:0032549	ribonucleoside binding	9.156e-03	EIF2AK2 MX1 OAS1 OAS3 GBP1 OASL OAS2
	GO:0001882	nucleoside binding	9.363e-03	EIF2AK2 MX1 OAS1 OAS3 GBP1 OASL OAS2
	GO:0017076	purine nucleotide binding	1.032e-02	EIF2AK2 MX1 OAS1 OAS3 GBP1 OASL OAS2
	GO:0044822	poly(A) RNA binding	5.088e-02	EIF2AK2 HERC5 IFIT2 OASL
CC	GO:0048471	perinuclear region of cytoplasm	3.888e-03	EIF2AK2 MX1 HERC5 OAS2
	GO:0005829	cytosol	9.919e-06	EIF2AK2 OAS1 OAS3 IRF7 HERC5 ISG15 OAS2 MX1 USP18 IFIT2 OTOF IFIT3 GBP1 IFIT1 OASL XAF1
	GO:0005739	mitochondrion	4.810e-03	OAS1 RSAD2 IFI27 IFIT3 IFI6 XAF1 OAS2
	GO:0005783	endoplasmic reticulum	1.583e-02	MX1 OAS1 IFIT2 RSAD2 OTOF OAS2
	GO:0012505	endomembrane system	4.168e-03	SAMD9L OAS1 SERPING1 IRF7 RSAD2 IFITM3 OAS2 MX1 IFIT2 OTOF IFI27 GBP1
	GO:0005737	cytoplasm	1.488e-02	SERPING1 RSAD2 ISG15 USP18 OTOF IFI27 IFIT3 OASL SAMD9L OAS1 OAS3 HERC5 IFI44 IFI6 IRF7 OAS2 GBP1 IFIT1 EIF2AK2 IFITM3 MX1 IFIT2 IFI44L XAF1
BP	GO:0035455	response to interferon-alpha	1.907e-12	IFITM3 IFIT3 OAS1 IFIT2 EIF2AK2
	GO:0071357	cellular response to type I interferon	2.702e-30	IFIT1 OAS3 MX1 USP18 IFIT2 IRF7 OAS1 OAS2 IFITM3 OASL IFIT3 XAF1 ISG15 RSAD2 IFI27 IFI6
	GO:0060337	type I interferon signaling pathway	2.702e-30	IFIT1 OAS3 MX1 USP18 IFIT2 IRF7 OAS1 OAS2 IFITM3 OASL IFIT3 XAF1 ISG15 RSAD2 IFI27 IFI6
	GO:0045071	negative regulation of viral genome replication	2.204e-18	OAS1 EIF2AK2 IFIT1 OAS3 MX1 IFITM3 OASL ISG15 RSAD2
	GO:0034340	response to type I interferon	3.905e-30	IFIT1 OAS3 MX1 USP18 IFIT2 IRF7 OAS1 OAS2 IFITM3 OASL IFIT3 XAF1 ISG15 RSAD2 IFI27 IFI6
	GO:0045069	regulation of viral genome replication	9.301e-17	EIF2AK2 IFIT1 OAS3 MX1 OAS1 IFITM3 OASL ISG15 RSAD2
	GO:0048525	negative regulation of viral process	4.916e-16	EIF2AK2 IFIT1 OAS3 MX1 OAS1 IFITM3 OASL ISG15 RSAD2
	GO:0019079	viral genome replication	9.276e-16	EIF2AK2 IFIT1 OAS3 MX1 OAS1 IFITM3 OASL ISG15 RSAD2
	GO:0060333	interferon-gamma-mediated signaling pathway	1.854e-10	OAS3 GBP1 IRF7 OAS1 OAS2 OASL
	GO:0051607	defense response to virus	6.646e-09	HLA-DPA1 GBP2 IFNGR1 VCAM1 HLA-DRA CCL22 ICAM1

**Table 2:** Top 10 KEGG pathways and their corresponding *P* values and genes.

Pathway ID	Description	*P* value	Genes
hsa05160	Hepatitis C	2.02329*e* − 11	IFIT1 IRF7 MX1 OAS1 OAS2 OAS3 EIF2AK2 RSAD2
hsa05164	Influenza A	2.60307*e* − 09	IRF7 MX1 OAS1 OAS2 OAS3 EIF2AK2 RSAD2
hsa05162	Measles	3.6267*e* − 08	IRF7 MX1 OAS1 OAS2 OAS3 EIF2AK2
hsa05169	Epstein-Barr virus infection	3.37146*e* − 07	IRF7 OAS1 OAS2 OAS3 EIF2AK2 ISG15
hsa04621	NOD-like receptor signaling pathway	5.95267*e* − 06	GBP1 IRF7 OAS1 OAS2 OAS3
hsa05165	Human papillomavirus infection	0.000110032	MX1 EIF2AK2 OASL ISG15 HES4
hsa05168	Herpes simplex virus 1 infection	0.000742976	IRF7 OAS1 OAS2 OAS3 EIF2AK2
hsa04622	RIG-I-like receptor signaling pathway	0.005431097	IRF7 ISG15
hsa05167	Kaposi sarcoma-associated herpesvirus infection	0.037274554	IRF7 EIF2AK2
hsa05203	Viral carcinogenesis	0.041244055	IRF7 EIF2AK2

**Table 3:** Topological result exploration for top 20 genes.

Hub gene	Degree	Stress	Closeness	Betweenness	Eccentricity	Clustering coefficient
MX1	23	20	23	1.09235	1	0.96047
IFIT1	23	20	23	1.09235	1	0.96047
ISG15	23	20	23	1.09235	1	0.96047
RSAD2	23	20	23	1.09235	1	0.96047
IFI44L	23	20	23	1.09235	1	0.96047
IFI44	23	20	23	1.09235	1	0.96047
IFIT3	23	20	23	1.09235	1	0.96047
OAS2	23	20	23	1.09235	1	0.96047
OAS1	23	20	23	1.09235	1	0.96047
IFI6	23	20	23	1.09235	1	0.96047
XAF1	23	20	23	1.09235	1	0.96047
IFIT2	23	20	23	1.09235	1	0.96047
GBP1	23	20	23	1.09235	1	0.96047
OAS3	23	20	23	1.09235	1	0.96047
HERC5	22	14	22.5	0.75624	0.5	0.9697
IRF7	22	10	22.5	0.51637	0.5	0.97835
OASL	22	10	22.5	0.51637	0.5	0.97835
IFI27	22	14	22.5	0.77598	0.5	0.9697
EIF2AK2	22	10	22.5	0.51637	0.5	0.97835
LY6E	21	10	22	0.55833	0.5	0.97619

**Table 4:** Candidate drug for pSS with fatigue.

Name of drugs	*P* value	Adjusted *P* value	Target genes
Acetohexamide PC3 UP	3.47*e* − 23	8.16*e* − 21	RSAD2; OAS1; OAS2; MX1; IFI6; IFI44; IFIT1; IFI44L
Suloctidil HL60 UP	2.19*e* − 22	2.57*e* − 20	RSAD2; OAS1; OAS2; MX1; IFI6; IFI44; ISG15; IFIT1; IFI44L; IFIT3
Prenylamine HL60 UP	3.46*e* − 21	2.71*e* − 19	OAS1; MX1; IFI6; IFI44; ISG15; IFIT1; IFI44L; IFIT3
Chlorophyllin CTD 00000324	9.59*e* − 19	5.64*e* − 17	OAS1 OAS2 MX1 IFI6 ISG15 IFIT1 IFIT3
Terfenadine HL60 UP	1.81*e* − 16	8.52*e* − 15	OAS1 MX1 IFI6 IFI44 ISG15 IFIT1 IFIT3
3′-Azido-3′-deoxythymidine CTD 00007047	6.05*e* − 13	2.37*e* − 11	RSAD2 OAS1 OAS2 MX1 IFI6 ISG15 IFIT1 IFI44L
Clioquinol PC3 UP	1.79*e* − 11	6.00*e* − 10	OAS1 MX1 IFI44 IFIT1 IFIT3
Etoposide HL60 UP	2.92*e* − 11	8.56*e* − 10	OAS1 MX1 IFI6 IFI44 ISG15 IFIT1
Testosterone enanthate CTD 00000155	1.03*e* − 09	2.68*e* − 08	RSAD2 OAS2 MX1 IFI6 ISG15 IFI44L IFIT3
Gadodiamide hydrate CTD 00002623	3.85*e* − 09	9.05*e* − 08	RSAD2 IFIT1 IFI44L IFIT3

## Data Availability

The dataset supporting the conclusions of this article is available in the UK registry of primary Sjögren's syndrome repository and in the hyperlink (https://www.ncbi.nlm.nih.gov/geo/geo2r/?acc=GSE66795).
